# Macrophage-derived mir-100-5p orchestrates synovial proliferation and inflammation in rheumatoid arthritis through mTOR signaling

**DOI:** 10.1186/s12951-024-02444-1

**Published:** 2024-04-22

**Authors:** Huan Liu, Yuehong Chen, Yupeng Huang, Ling Wei, Jingjing Ran, Qianwei Li, Yunru Tian, Zhongling Luo, Leiyi Yang, Hongjiang Liu, Geng Yin, Qibing Xie

**Affiliations:** 1 Department of Rheumatology and Immunology, West China Hospital, Sichuan University, Chengdu, 610041 China; 2 Department of General Practice, West China Hospital, General Practice Medical Center, Sichuan University, Chengdu, 610041 China; 3 Hospital of Chengdu Office of People’s Government of Tibetan Autonomous region, Chengdu, 610041 China

**Keywords:** Rheumatoid arthritis, Macrophages, Extracellular vesicles, miR-100-5p, mTOR

## Abstract

**Background:**

Rheumatoid arthritis (RA) is a chronic autoimmune disorder characterized by synovial inflammation, causing substantial disability and reducing life quality. While macrophages are widely appreciated as a master regulator in the inflammatory response of RA, the precise mechanisms underlying the regulation of proliferation and inflammation in RA-derived fibroblast-like synoviocytes (RA-FLS) remain elusive. Here, we provide extensive evidence to demonstrate that macrophage contributes to RA microenvironment remodeling by extracellular vesicles (sEVs) and downstream miR-100-5p/ mammalian target of rapamycin (mTOR) axis.

**Results:**

We showed that bone marrow derived macrophage (BMDM) derived-sEVs (BMDM-sEVs) from collagen-induced arthritis (CIA) mice (cBMDM-sEVs) exhibited a notable increase in abundance compared with BMDM-sEVs from normal mice (nBMDM-sEVs). cBMDM-sEVs induced significant RA-FLS proliferation and potent inflammatory responses. Mechanistically, decreased levels of miR-100-5p were detected in cBMDM-sEVs compared with nBMDM-sEVs. miR-100-5p overexpression ameliorated RA-FLS proliferation and inflammation by targeting the mTOR pathway. Partial attenuation of the inflammatory effects induced by cBMDM-sEVs on RA-FLS was achieved through the introduction of an overexpression of miR-100-5p.

**Conclusions:**

Our work reveals the critical role of macrophages in exacerbating RA by facilitating the transfer of miR-100-5p-deficient sEVs to RA-FLS, and sheds light on novel disease mechanisms and provides potential therapeutic targets for RA interventions.

**Graphical abstract:**

**Figure:**
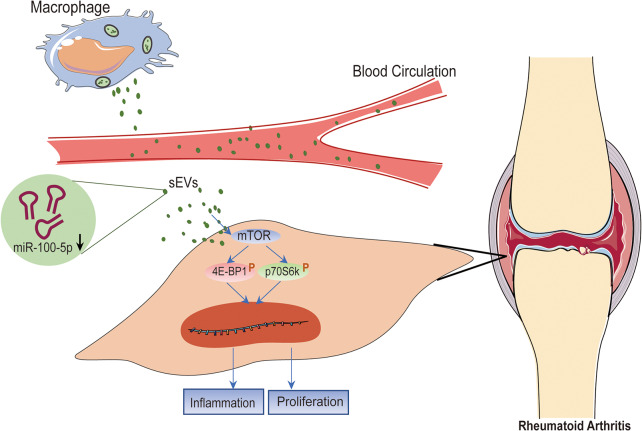

**Supplementary Information:**

The online version contains supplementary material available at 10.1186/s12951-024-02444-1.

## Introduction

Rheumatoid arthritis (RA) is a chronic and widespread autoimmune disease characterized by progressive synovitis and joint degradation [1, 2], with a prevalence rate of approximately 246.6/100,000 [3]. As one of the most common and disabling diseases encountered in clinical practice, the exact etiology and pathogenesis of RA remain elusive [1]. A hallmark of RA is the reorganization of synovium, encompassing the expansion of fibroblast-like synoviocytes (FLS) and the infiltration of immune cells, including macrophages, T cells, and B cells [4]. Activated FLS, in particular, play a crucial role as pathogenic cells by inducing cartilage destruction and synovitis through the secretion of inflammatory cytokines and extracellular matrix components. Despite the recognition of the critical interplay between pro-inflammatory immune cells and activated FLS in the synovial inflammatory microenvironment, the intricate mechanisms governing FLS activation remain poorly understood [5]. Therefore, unraveling the molecular underpinnings and pathogenesis of RA and identifying new therapeutic targets have become pivotal pursuits in the realm of rheumatic disease research.

Macrophages, as pivotal immune and inflammatory cells, have emerged as key players in the pathogenesis of RA [6]. Recent advancements in high-throughput sequencing technologies, such as single-cell RNA sequencing and spatial transcriptomics, have unveiled the remarkable heterogeneity of macrophages within the RA synovium [7]. These findings also underscore the significance of macrophages in promoting RA-derived FLS (RA-FLS) proliferation and secretion of inflammatory mediators, including interleukin 1β (IL-1β), interleukin 6 (IL-6), interleukin 8 (IL-8), and tumor necrosis factor α (TNF-α), ultimately contributing to synovial hyperplasia, cartilage and bone erosion, joint inflammation and stiffness [7–9]. Yet, precise mechanisms underlying the intricate crosstalk between macrophages and RA-FLS are still not fully understood.

Previous studies have highlighted the ability of macrophages to modulate effector cell functions through the transfer of extracellular vesicles (EVs) in various diseases [10, 11]. Among these, small extracellular vesicles (sEVs) have garnered significant attention. As natural nanoscale vesicles with diameters smaller than 200 nm, sEVs are actively secreted by living cells and serve as vehicles for transmitting “molecular messages” to adjacent or distant cells via humoral circulation [12]. These sEVs encapsulate a diverse cargo of DNA, mRNA, miRNA, proteins, and lipids from the donor cells, facilitating their communication with recipient cells [13, 14]. Furthermore, sEVs boast low immunogenicity and possess the ability to traverse the blood-brain barrier, making them ideal non-cellular carriers of molecular information [15]. miRNAs, a class of short non-coding RNAs, have emerged as prominent regulators of gene expression at the post-transcriptional level [16, 17]. The inclusion of miRNAs in sEVs confers protection against enzymatic degradation in bodily fluids, enabling them to reach target cells and exert robust biological regulatory effects [18]. Notably, certain miRNAs carried by sEVs have been implicated in the regulation of the synovial microenvironment in RA, emphasizing their capacity to mediate stable communications and modulate the RA state [19–22].

In this study, we explored the secretion of sEVs encapsulating aberrantly expressed miRNAs from macrophages exposed to the pathological milieu of RA. These sEVs targeted FLS via humoral circulation, promoting excessive proliferation and inflammatory expression. Diminished levels of miR-100-5p were observed in arthritic sEVs, and its reintroduction alleviated proliferation and inflammation by targeting the mammalian target of rapamycin (mTOR) signaling. Overall, our study reveals the role of macrophages in exacerbating RA through the transfer of miR-100-5p-deficient sEVs to FLS, providing insights for therapeutic interventions.

## Results

### Assessment of BMDM-sEVs from RA animals

**Fig. 1:**
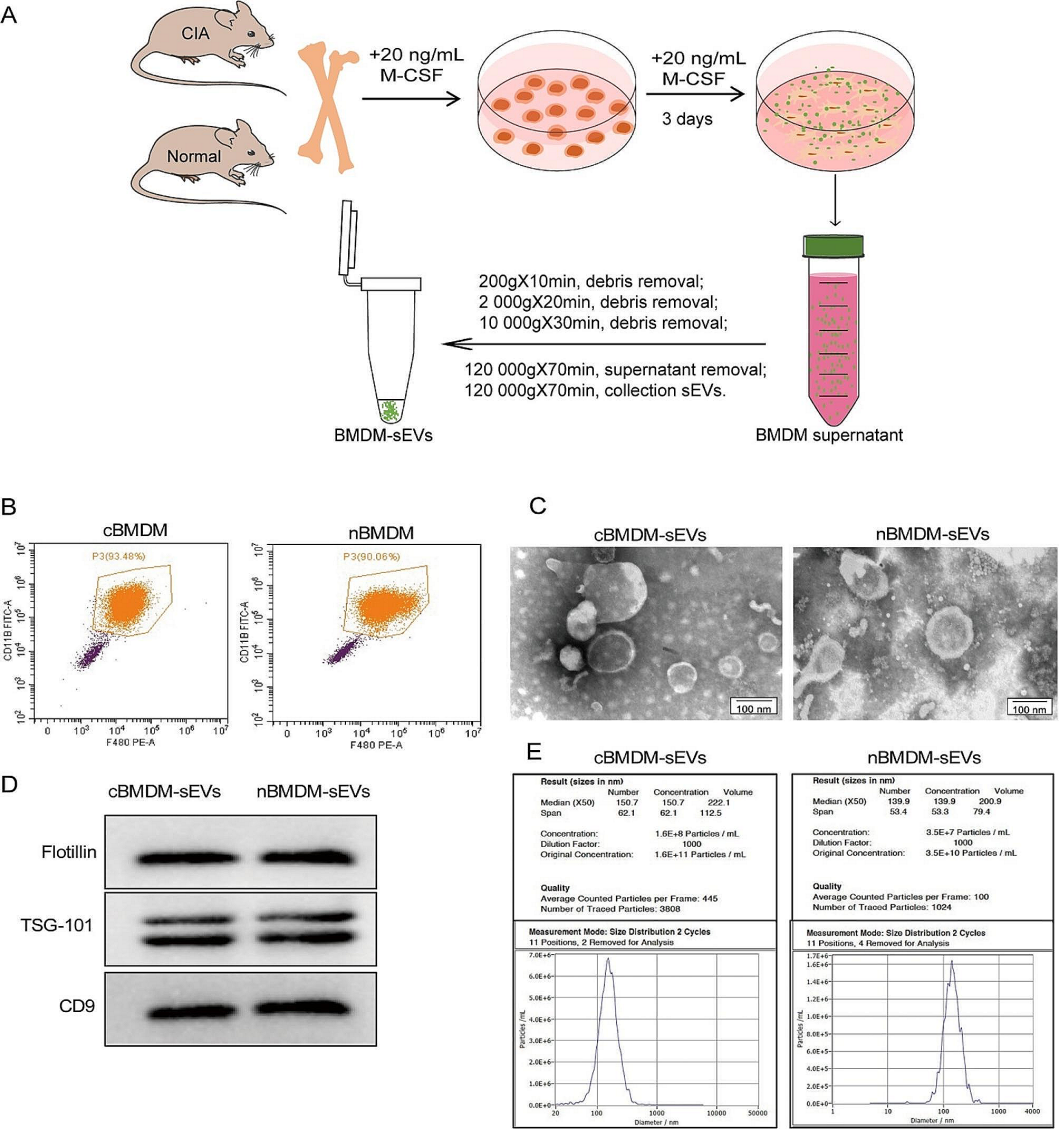
Identification of BMDMs and their sEVs. **(A)** Extraction process of BMDM-sEVs. **(B)** BMDMs surface makers F4/80 and CD11b analyzed by flow cytometry. **(C)** Morphology of sEVs determined by TEM. **(D)** sEVs surface markers Flotillin, TSG101, and CD9 examined by WB. **(E)** Diameter and concentration of sEVs examined by NTA

First, we extracted bone marrow cells from both the collagen-induced arthritis (CIA) model and normal mice, followed by in vitro differentiation into bone marrow derived macrophage (BMDM) (Fig. 1A). The BMDM population was characterized using flow cytometry (Fig. 1B), showing that approximately 90% of the cells exhibited positive expression of F4/80 and CD11b, confirming their macrophage phenotype. Subsequently, transmission electron microscopy **(**TEM) analysis showed that the obtained BMDM-derived sEVs (BMDM-sEVs) possessed the characteristic bilayer lipid membrane structure and an average size of approximately 150 nm (Fig. 1C). Western blot (WB) analysis confirmed the presence of specific sEV markers, including TSG-101, Flotillin, and CD9 (Fig. 1D). Nanoparticle tracking analysis (NTA) further substantiated that the size of sEVs in both groups was around 150 nm, while the number of sEVs secreted from CIA mice derived-BMDM-sEVs (cBMDM-sEVs) was approximately three times higher compared with those from normal mice derived BMDM-sEVs (nBMDM-sEVs) (Fig. 1E). These results collectively validate the successful isolation of BMDM-sEVs from both the CIA and normal groups, indicating that cBMDM exhibit an enhanced capacity for sEV secretion compared with nBMDM with an equivalent number of macrophages.

### cBMDM-sEVs effectively stimulate the proliferation and inflammation of RA-FLS

**Fig. 2:**
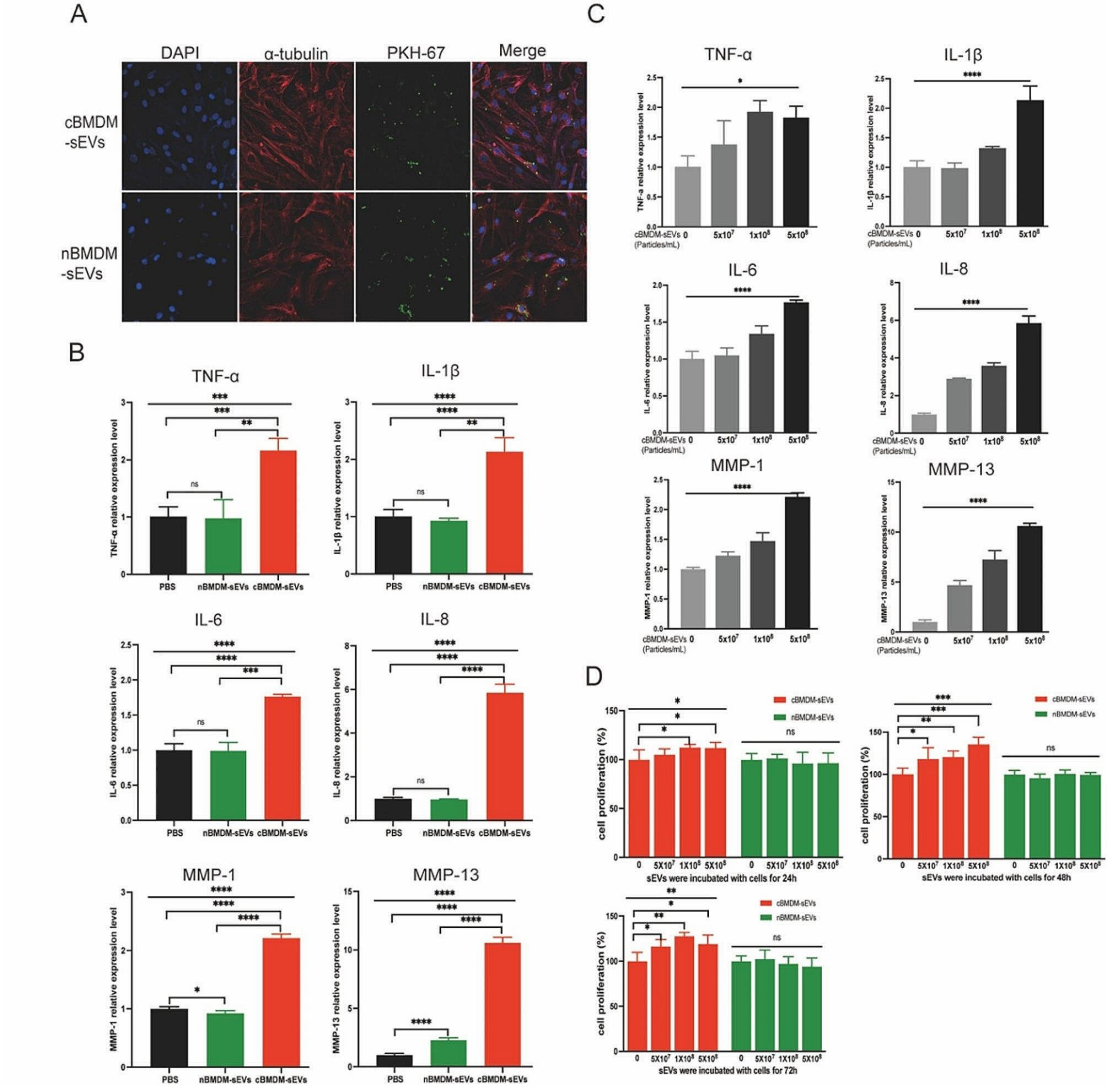
Effects of cBMDM-sEVs on proliferation and inflammation of RA-FLS in vitro. **(A)** BMDM-sEVs internalized by RA-FLS. **(B)** mRNA level of inflammatory cytokines in RA-FLS co-cultured with PBS, cBMDM-sEVs, or nBMDM-sEVs, as determined by qRT-PCR. **(C)** mRNA level of inflammatory cytokines in RA-FLS co-cultured with gradient concentration of cBMDM-sEVs. **(D)** Proliferation of RA-FLS co-cultured with PBS, cBMDM-sEVs, or nBMDM-sEVs, as determined by CCK8. Data are presented as mean ± SD, *p < 0.05, **p < 0.01, ***p < 0.001, ****p < 0.0001

Next, we investigated the impact of BMDM-sEVs from different sources on RA-FLS, with a primary focus on elucidating whether these sEVs could effectively penetrate the FLS. Thus, both cBMDM-sEVs and nBMDM-sEVs were labeled with PKH67 and co-cultured with RA-FLS for 12 h. Results showed that both types of sEVs were successfully internalized into the cytoplasm of RA-FLS, with no evidence of nuclear entry (Fig. 2A). Subsequently, cBMDM-sEVs (5 × 10^8^ particles/mL), nBMDM-sEVs (5 × 10^8^ particles/mL), and equal volumes of phosphate buffer saline (PBS) were separately co-cultured with RA-FLS for 24 h, and the levels of inflammatory factors as well as members of the matrix metalloproteinases (MMPs) family were assessed. Remarkably, cBMDM-sEVs elicited a significant increase in the expression of TNF-α, IL-1β, IL-6, IL-8, MMP-1, and MMP-13 compared with nBMDM-sEVs and the PBS control (Fig. 2B and S1), but the expression of MMP-3 and MMP-9 was no significant difference among three groups. Notably, the expression levels of these factors (except MMP-3 and MMP-9) demonstrated a positive correlation with the concentration of cBMDM-sEVs when different concentrations were co-cultured with RA-FLS (Fig. 2C and S2). Moreover, RA-FLS co-cultured with various concentrations of cBMDM-sEVs and nBMDM-sEVs were examined for cell proliferation after 24, 48, and 72 h (Fig. 2D). Results showed that cBMDM-sEVs prominently stimulated the proliferation of RA-FLS when compared to both the PBS control and the nBMDM-sEVs group. Collectively, these findings provide compelling evidence that cBMDM-sEVs play a crucial role in promoting the proliferation and inflammation of RA-FLS.

### cBMDM-sEVs exacerbated arthritis in RA animals

**Fig. 3:**
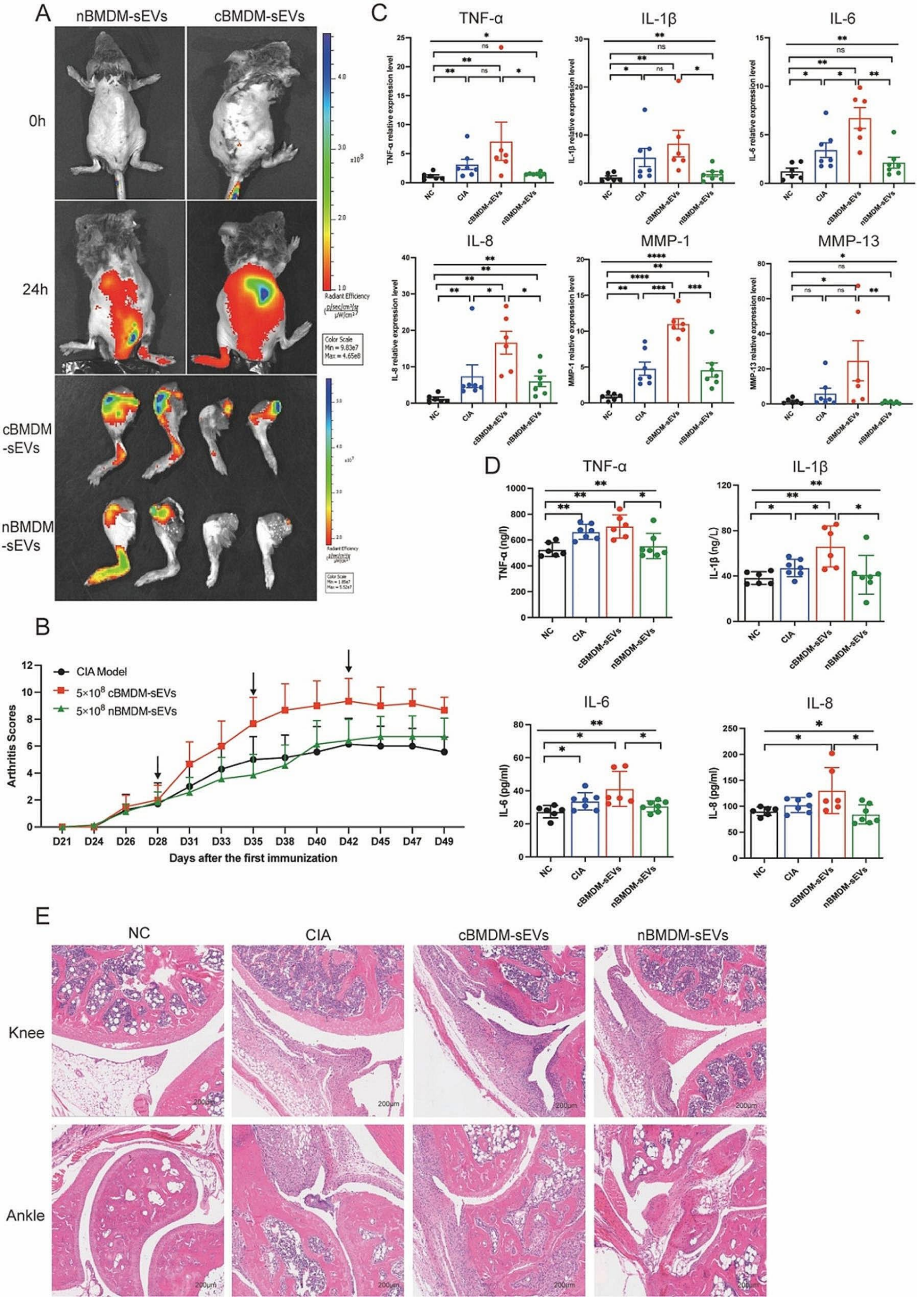
cBMDM-sEVs aggravated arthritis in CIA models. **(A)** cBMDM-sEVs and nBMDM-sEVs labeled with DiR, and fluorescence images obtained at 0 and 24 h after intravenous injection of the DIR-labeled BMDM-sEVs into CIA mice. **(B)** Arthritis score in three groups. **(C)** mRNA expression of TNF-α, IL-1β, IL-6, IL-8, MMP-1, and MMP-13 in ankle joint of four groups. **(D)** Levels of TNF-α, IL-1β, IL-6, and IL-8 in serum of four groups. **(E)** H&E staining of knee and ankle joints in CIA mice. Each group consists of 6–7 mice. Data are presented as mean ± SD, *p < 0.05, **p < 0.01, ***p < 0.001, ****p < 0.0001

The involvement of EVs in cell-to-cell communication and their ability to traverse distant tissues via bodily fluid circulation have been widely documented [23–25]. To elucidate the potential role of BMDM-sEVs in the context of CIA mice, we labeled cBMDM-sEVs and nBMDM-sEVs with DiR and administered them to CIA mice intravenously. Results showed that both cBMDM-sEVs and nBMDM-sEVs were observed to effectively reach the inflamed joints after 24 h, signifying their ability to access the inflammatory site via circulation in the body fluids (Fig. 3A). Next, we examined the impact of cBMDM-sEVs and nBMDM-sEVs on the CIA models. Notably, weekly injections of cBMDM-sEVs (5 × 10^8^ particles) via the tail vein resulted in a rapid aggravation of inflammation. In contrast, mice receiving an equivalent amount of nBMDM-sEVs did not exhibit aggravated inflammation at the early stage (Fig. 3B). While with prolonged injection accumulation, nBMDM-sEVs also contributed to increased inflammation, albeit to a lesser extent than that caused by cBMDM-sEVs. In addition, when cBMDM-sEVs and nBMDM-sEVs were injected at a lower dose (5 × 10^7^ particles), no significant differences in joint inflammation were observed among the three groups (Figure S3).

Furthermore, quantitative real-time PCR (qRT-PCR) analysis of ankle joint tissue revealed dramatically higher mRNA levels of TNF-α, IL-1β, IL-6, IL-8, MMP-1, and MMP-13 in the cBMDM-sEVs group compared with the PBS and nBMDM-sEVs groups (Fig. 3C). ELISA analysis of serum further confirmed these findings, demonstrating significant elevations in the levels of TNF-α, IL-1β, IL-6, and IL-8 in the cBMDM-sEVs group compared with NC and nBMDM-sEVs group. However, it’s important to note that only IL-1β exhibited a significant increase in the cBMDM-sEVs group when compared with the CIA group (Fig. 3D). Finally, histological examination of knee and ankle joints using hematoxylin and eosin staining (H&E) revealed more severe synovial hyperplasia, pannus formation, inflammatory cell infiltration, and bone erosion in the cBMDM-sEVs group compared with the PBS and nBMDM-sEVs groups. All these results suggest that BMDM-sEVs, particularly those derived from CIA mice, play a pivotal role in exacerbating arthritis in the CIA mouse model.

### Identification of differentially expressed miRNAs in cBMDM-sEVs and nBMDM-sEVs

**Fig. 4:**
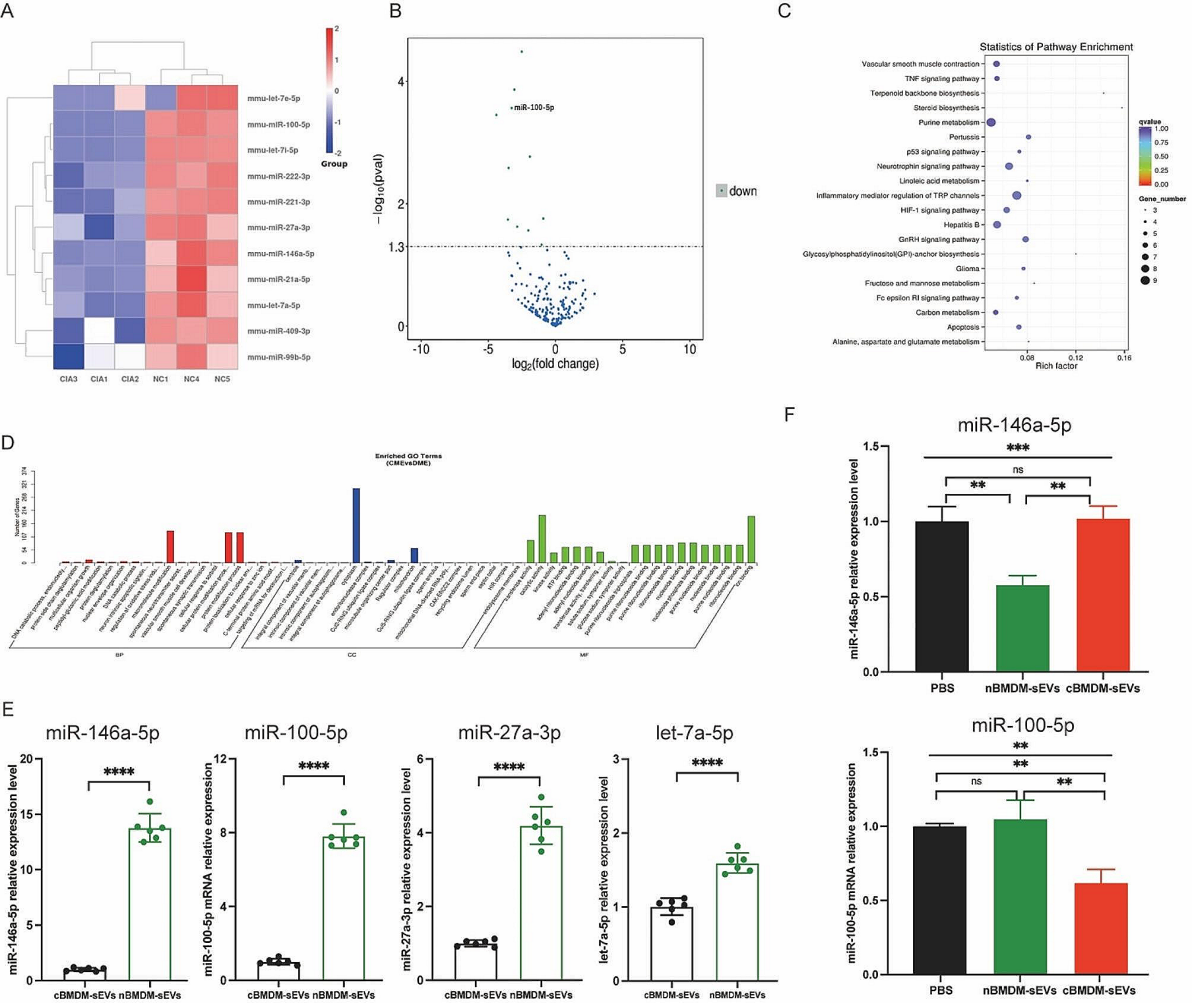
Expression of miRNA in cBMDM-sEVs and nBMDM-sEVs. **(A)** Heatmap analysis of miRNA in cBMDM-sEVs and nBMDM-sEVs (*n* = 3). **(B)** Volcano plot of differentially expressed miRNA between cBMDM-sEVs and nBMDM-sEVs. **(C)** KEGG pathway analyses of differentially expressed miRNA between cBMDM-sEVs and nBMDM-sEVs. **(D)** GO analyses of differentially expressed miRNA between cBMDM-sEVs and nBMDM-sEVs. **(E)** mRNA expression of miR-146a-5p, miR-100-5p, miR-27a-3p, and let-7a-5p in cBMDM-sEVs and nBMDM-sEVs by qRT-PCR. **(F)** mRNA expression of miR-146a-5p and miR-100-5p in RA-FLS co-cultured with PBS, cBMDM-sEVs or nBMDM-sEVs, respectively. Data are presented as mean ± SD, *p < 0.05, **p < 0.01, ***p < 0.001, ****p < 0.0001

To better understand the potential mechanisms underlying the selective transport of miRNAs, we embarked on an analysis of miRNA profiles in BMDM-sEVs derived from CIA and normal mice. Strikingly, a subset of miRNAs, including miR-146a-5p, miR-100-5p, miR-27a-3p, let-7a-5p, miR-21a-5p, miR-99b-5p, let-7e-5p, miR-222-3p, miR-409-3p, let-7i-5p, and miR-221-3p, exhibited significant downregulation in cBMDM-sEVs when compared with nBMDM-sEVs (Fig. 4A-D). Interestingly, our analysis exclusively identified miRNAs with reduced expression in cBMDM-sEVs, with no miRNAs displaying upregulated expression. To validate the differential miRNA expression in cBMDM-sEVs and nBMDM-sEVs, we performed qRT-PCR analysis on selected miRNAs (miR-146a-5p, miR-100-5p, miR-27a-3p, and let-7a-5p) extracted from cBMDM-sEVs and nBMDM-sEVs. Results showed higher expression levels of these selected miRNAs in cBMDM-sEVs compared with nBMDM-sEVs, albeit with some variation (Fig. 4E). Among these miRNAs, miR-146a-5p and miR-100-5p displayed distinct expression patterns between the cBMDM-sEVs and nBMDM-sEVs, and were selected for further investigations.

Next, we asked whether BMDMs from distinct sources transferred varying miRNA levels to RA-FLS via sEVs, thereby influencing the biological function of RA-FLS. We found that the expression of miR-146a-5p in RA-FLS co-cultured with nBMDM-sEVs was notably lower than that in RA-FLS co-cultured with cBMDM-sEVs or PBS (Fig. 4F). In contrast, the expression of miR-100-5p in RA-FLS co-cultured with cBMDM-sEVs exhibited a significant reduction compared with the PBS and nBMDM-sEVs co-culture groups. These results underscore the potential of cBMDM to promote RA-FLS proliferation and inflammation by releasing sEVs with diminished miR-100-5p levels, while miR-146a-5p does not seem to play a comparable role in this context.

### Suppression of proliferation and inflammation of RA-FLS via miR-100-5p

**Fig. 5:**
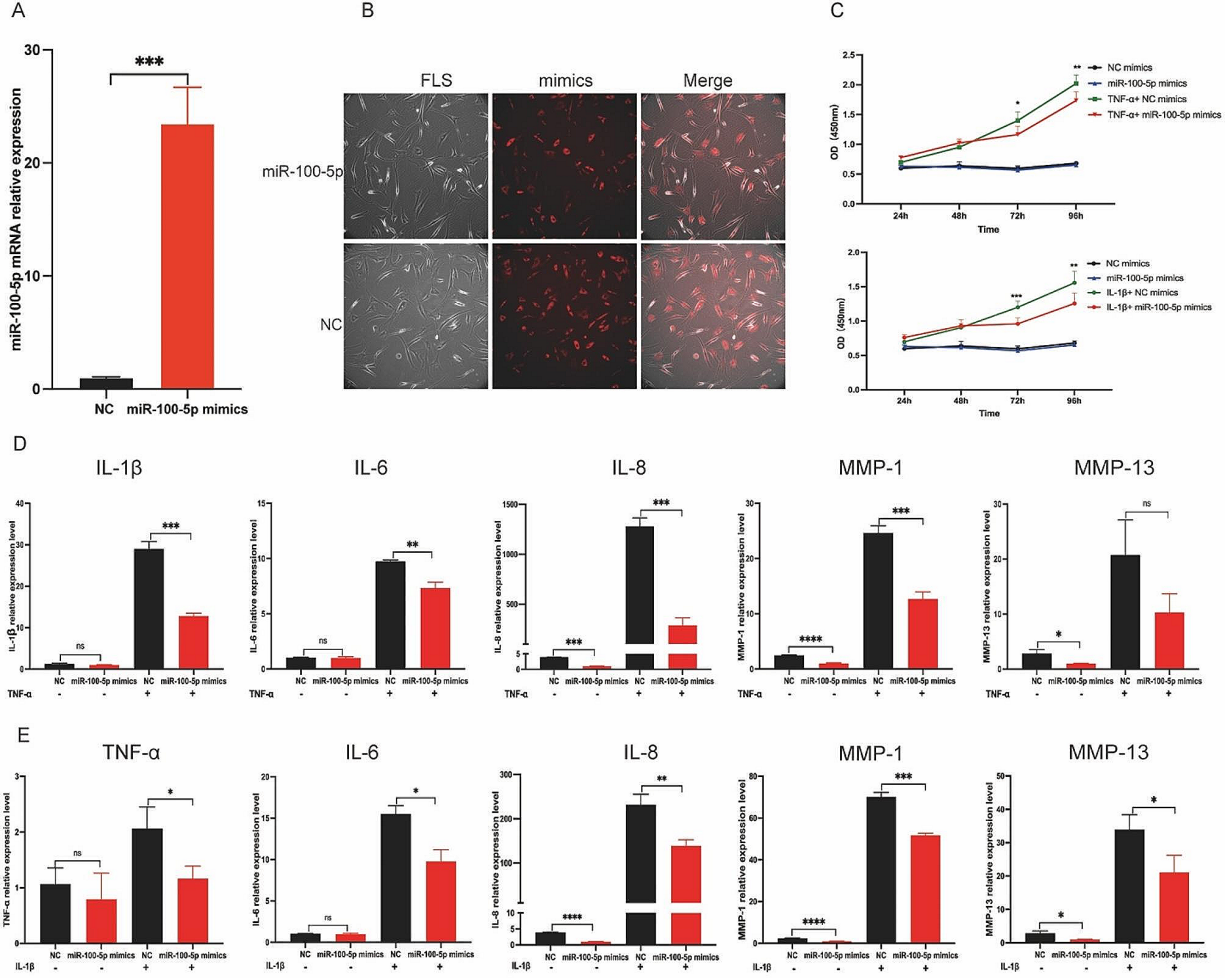
Effect of miR-100-5p on inflammation and proliferation of RA-FLS. **(A)** mRNA expression of miR-100-5p in RA-FLS transfected with NC mimics or miR-100-5p mimics analyzed by qRT-PCR. **(B)** Images of RA-FLS transfected with NC mimics-cy3 or miR-100-5p mimics-cy3. **(C)** Proliferation of RA-FLS transfected with NC or miR-100-5p mimics and stimulated with or without 10 ng/mL IL-1β or 25 ng/mL TNF-α at 24, 48, 72, and 96 h by CCK8. **(D)** mRNA expression of IL-1β, IL-6, IL-8, MMP-1, and MMP-13 in RA-FLS transfected with NC or miR-100-5p mimics and stimulated with or without 25 ng/mL TNF-α. **(E)** mRNA expression of TNF-α, IL-6, IL-8, MMP-1, and MMP-13 in RA-FLS transfected with NC or miR-100-5p mimics and stimulated with or without 10 ng/mL IL-1β. Data are presented as mean ± SD, *p < 0.05, **p < 0.01, ***p < 0.001, ****p < 0.0001

To elucidate the impact of miR-100-5p on RA-FLS, we conducted transfection experiments using miR-100-5p mimics, negative control (NC) mimics, miR-100-5p inhibitors, or NC inhibitors in RA-FLS, and verified the transfection efficiencies through qRT-PCR and immunofluorescence (IF) analyses (Fig. 5A and B, and Figure S4). The influence of miR-100-5p overexpression on RA-FLS proliferation was investigated under the stimulation of either 10 ng/mL IL-1β or 25 ng/ mL TNF-α. Notably, examination at various time points (24, 48, 72, and 96 h) revealed that miR-100-5p exerted inhibitory effects on RA-FLS proliferation under the stimulation of IL-1β or TNF-α, particularly evident at 72- and 96-hour post-stimulation (Fig. 5C).

Then we analyzed the relative expressions of inflammatory factors (IL-1β, IL-6, IL-8, MMP-1, and MMP-13) in FLS transfected with miR-100-5p mimics or inhibitors under TNF-α or IL-1β stimulation conditions. Intriguingly, qRT-PCR analysis demonstrated a significant decrease in the relative expressions of IL-1β, IL-6, IL-8, MMP-1, and MMP-13 in RA-FLS transfected with miR-100-5p mimics under TNF-α stimulation (Fig. 5D). Conversely, the relative expressions of TNF-α, IL-6, IL-8, MMP-1, and MMP-13 were markedly increased in RA-FLS transfected with miR-100-5p inhibitors (Figure S5). Similarly, upon IL-1β stimulation, miR-100-5p mimic transfection led to suppression of TNF-α, IL-6, IL-8, MMP-1, and MMP-13 expressions (Fig. 5E), while miR-100-5p inhibitor transfection resulted in their upregulation (Figure S6). These observations confirm that miR-100-5p overexpression effectively inhibits the inflammatory response of RA-FLS stimulated by IL-1β or TNF-α, whereas low miR-100-5p expression promotes their inflammatory response and proliferation. Thus, our findings highlight the crucial role of miR-100-5p in regulating RA-FLS behavior in response to pro-inflammatory cytokines.

### Attenuation of arthritis in RA animals by miR-100-5p agomiR treatment

**Fig. 6:**
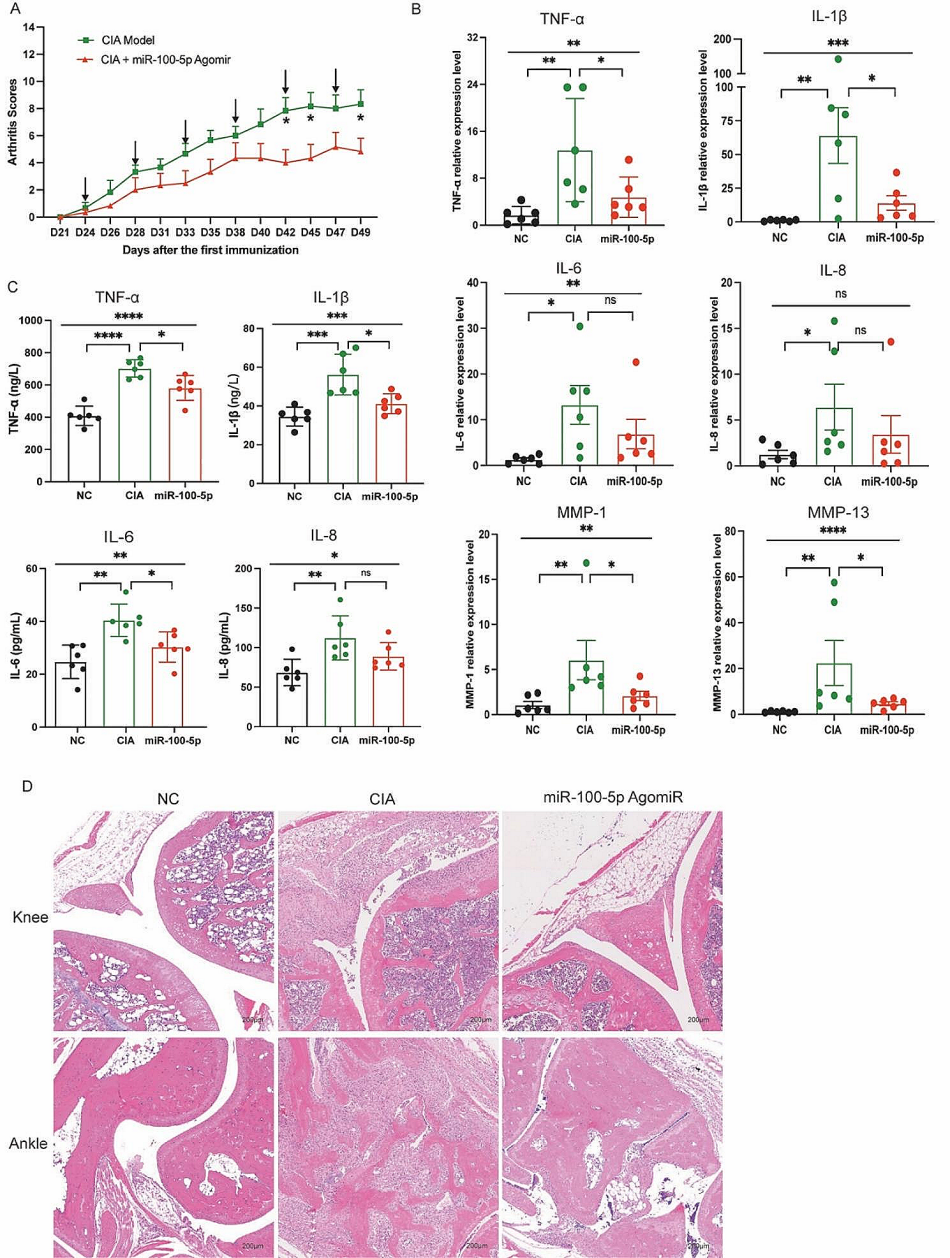
miR-100-5p attenuation arthritis of CIA mice. **(A)** Arthritis score in two groups. **(B)** mRNA expression of TNF-α, IL-1β, IL-6, IL-8, MMP-1, and MMP-13 in ankle joint of three groups. **(C)** Levels of TNF-α, IL-1β, IL-6, and IL-8 in serum of three groups. **(D)** H&E staining of knee and ankle joints of CIA and normal control mice. Data are presented as mean ± SD, *p < 0.05, **p < 0.01, ***p < 0.001, ****p < 0.0001

Building upon the above findings in vitro, we administered miR-100-5p agomiR, a chemically modified miR-100-5p agonist, into CIA mice through tail vein injection. Subsequent evaluation of arthritis progression revealed a notable reduction in the arthritis score in the miR-100-5p agomiR-treated group compared to the PBS-treated group (Fig. 6A). Moreover, analysis of mRNA relative expressions demonstrated a partial decrease in TNF-α, IL-1β, IL-6, IL-8, MMP-1, and MMP-13 in the miR-100-5p agomiR-treated CIA mice (Fig. 6B).

Additionally, the levels of TNF-α, IL-1β, IL-6, and IL-8 in the serum exhibited a partial reduction relative to the PBS-treated group (Fig. 6C). Histological examination of knee and ankle joint tissues further corroborated these findings, with HE staining revealing amelioration of synovial hyperplasia, pannus formation, inflammatory cell infiltration, and bone erosion in the miR-100-5p agomiR-treated group compared to the PBS group (Fig. 6D). Altogether, these results demonstrate the therapeutic potential of miR-100-5p agomiR in alleviating arthritis severity in CIA mice, further reinforcing the importance of miR-100-5p as a promising target for potential therapeutic interventions in rheumatoid arthritis.

### The impact of miR-100-5p on rheumatoid arthritis via mTOR signaling

**Fig. 7:**
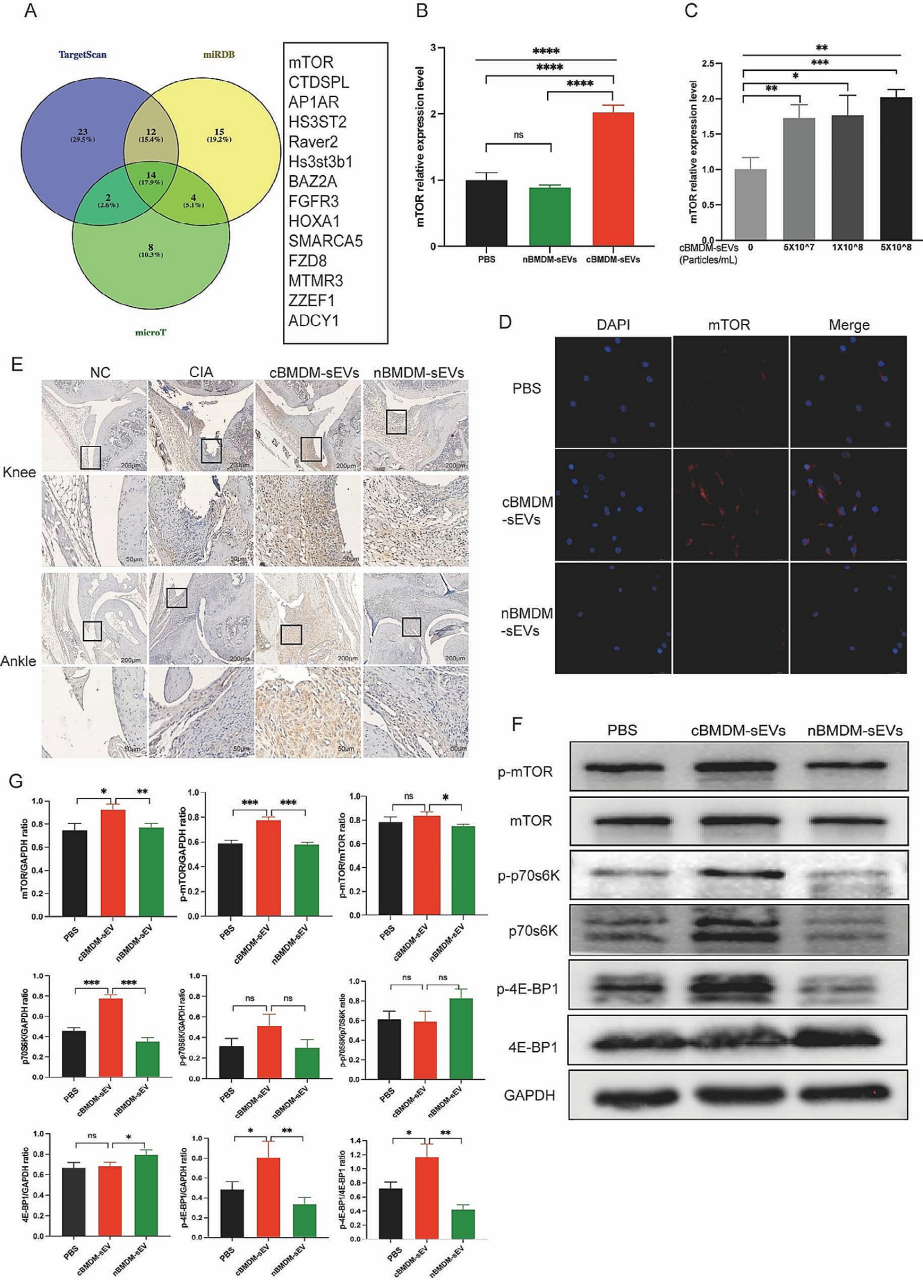
Effect of cBMDM-sEVs on proliferation and inflammation of synovium in vivo and in vitro by targeting mTOR and downstream signal pathway. (**A**) Venn diagram of the targeting genes of miR-100-5p predicted by TargetScan, miRDB, and microT. (**B**) mRNA expression of mTOR in RA-FLS cocultured with PBS, cBMDM-sEVs or nBMDM-sEVs, respectively. (**C**) mRNA expression of mTOR in RA-FLS cocultured with different concentrations of cBMDM-sEVs. (**D**) Representative fluorescence images of mTOR in RA-FLS cocultured with PBS, cBMDM-sEVs or nBMDM-sEVs. (**E**) mTOR expression in knee and ankle joints analyzed by IHC. (**F**-**G**) Protein expression of mTOR, p-mTOR, p70s6K, p-p70s6K, 4E-BP1, and p-4E-BP1 by WB. Data are presented as mean ± SD, *p < 0.05, **p < 0.01, ***p < 0.001, ****p < 0.0001

To elucidate the mechanisms by which miR-100-5p regulating the proliferation and inflammation of synovial cells, we employed TargetScan, miRDB, and microT bioinformatics platforms, and revealed mTOR as a potential target of miR-100-5p (Fig. 7A). Previous studies have already confirmed the binding of miR-100-5p to the 3’-UTR region of mTOR through double-luciferase reporter gene experiments [26–30], obviating the need for repeating this experiment. We assessed mTOR expression in RA-FLS co-cultured with distinct BMDM-sEVs at the mRNA (Fig. 7B), protein (Fig. 7F and G) and localization levels (Fig. 7D). We found that mTOR expression in RA-FLS co-cultured with cBMDM-sEVs was significantly higher compared to those co-cultured with nBMDM-sEVs and PBS. Furthermore, upon addition of various concentrations of cBMDM-sEVs, RA-FLS exhibited elevated mRNA expression of mTOR in contrast to the PBS group (*p* < 0.01) (Fig. 7C). Nevertheless, no significant correlation was observed between the mRNA expression levels of mTOR and the concentration gradient of cBMDM-sEVs.

**Fig. 8:**
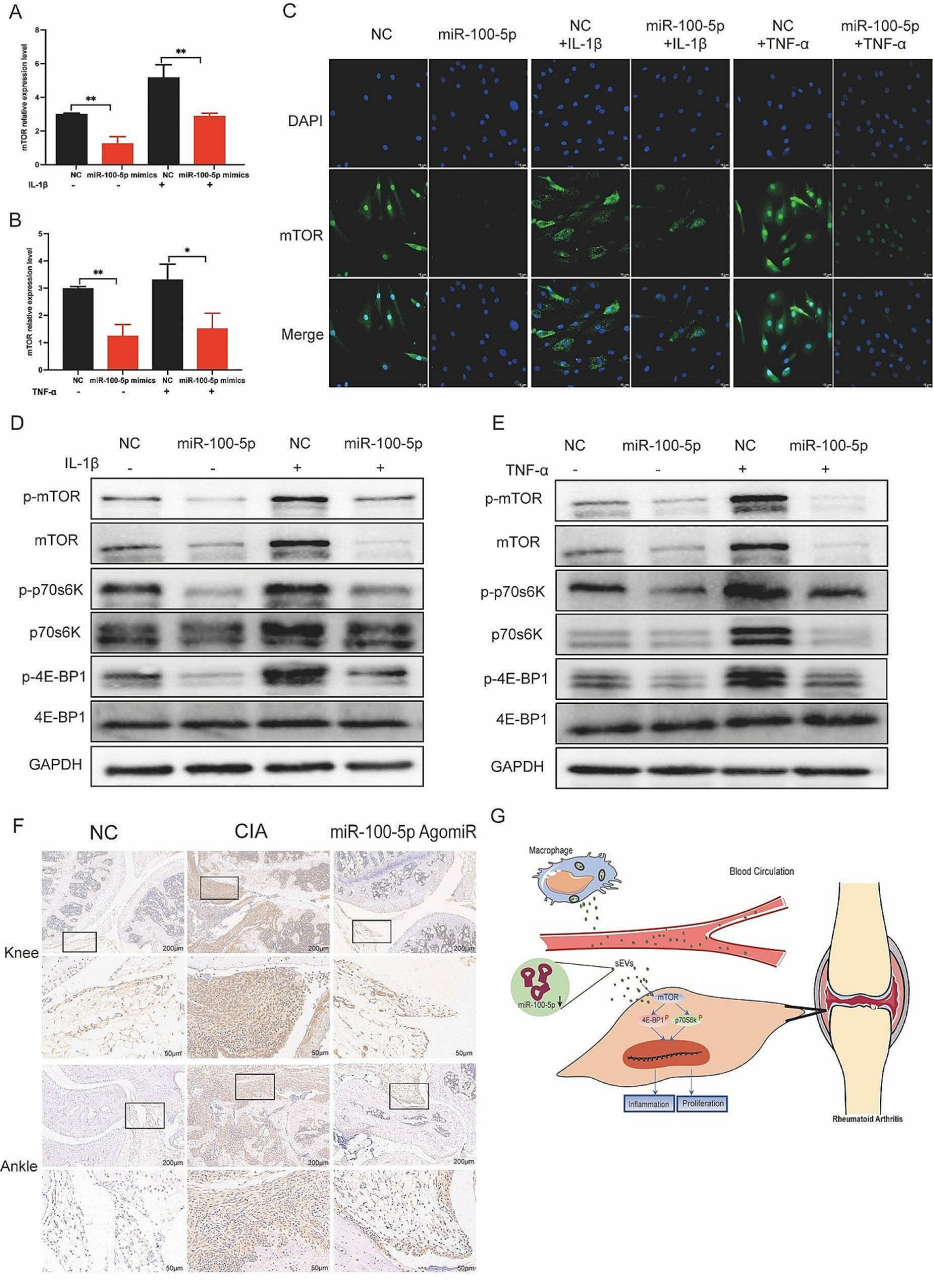
Effect of miR-100-5p on proliferation and inflammation of synovium in vivo and in vitro by inhibiting mTOR and downstream signal pathway. (**A**-**C**) Expression of mTOR in RA-FLS transfected with NC or miR-100-5p mimics and stimulated with TNF-α or IL-1β. (**D**-**E**) Protein expression of mTOR/p70s6K/4E-BP1 signal pathway by Western blot. (**F**) mTOR expression in knee and ankle joints by IHC. (**G**) Proposed model explaining how cBMDM-sEVs with low miR-100-5p levels promote synovitis via targeting mTOR signaling

Upon transfection of miR-100-5p mimics or inhibitors, we evaluated the expression of mTOR at both the mRNA and protein levels. Our results indicated that miR-100-5p mimics reduced mTOR expression (Fig. 8A-C), whereas miR-100-5p inhibitors increased it (Figure S7). All these data support the notion that miR-100-5p is a regulator of mTOR expression. Next, we examined mTOR’s downstream targets p70 ribosomal protein S6 kinase (p70s6K) and 4E-Binding protein 1(4E-BP1). Combining the results with Kyoto encyclopedia of genes and genomes (KEGG) pathway analysis, we hypothesized that mTOR influences the proliferation and inflammatory response of cells through the regulation of downstream p70s6K and 4E-BP1 activation and protein expression. To test this hypothesis, we assessed the expression of p70s6K, p-p70s6K, 4E-BP1, and p-4E-BP1 in RA-FLS co-cultured with BMDM-sEVs from different sources (Fig. 7F) and in RA-FLS transfected with miR-100-5p mimics or inhibitors (Fig. 8D and E).

Moreover, in vivo analyses demonstrated higher mTOR expression in the cBMDM-sEV group (Fig. 7E). Additionally, the expression of mTOR in the knee and ankle joints of CIA mice injected with miR-100-5p agomiR was assessed, showing significantly reduced mTOR expression in the joints of CIA mice treated with miR-100-5p agomiR (Fig. 8F). All these results underscore the significance of miR-100-5p regulating mTOR signaling in RA pathogenesis.

### Partial rescue of inflammatory effects of cBMDM-sEVs by miR-100-5p overexpression

To further investigate whether the inflammatory expression of RA-FLS induced by cBMDM-sEVs is associated with the downregulation of miR-100-5p in RA-FLS, we performed transfections of miR-100-5p mimics and NC mimics in RA-FLS. After 24 h, additional cBMDM-sEVs were introduced to the transfected FLS, followed by IL-1β or TNF-α stimulation, and cells were harvested after 24 h. qRT-PCR was conducted to assess the relative expression of TNF-α, IL-1β, IL-6, IL-8, MMP-1, and MMP-13 at the mRNA level.

RA-FLS transfected with NC mimics showed significantly increased expression of TNF-α, IL-6, IL-8, MMP-1, and MMP-13, confirming the successful induction of IL-1β stimulation (Figure S8A-E). When cBMDM-sEVs were added to RA-FLS co-cultured with NC mimics, the expression of these factors in RA-FLS was markedly elevated (*P* < 0.05) (Figures S8A-E). Conversely, RA-FLS transfected with miR-100-5p mimics exhibited reduced expression of TNF-α, IL-6, IL-8, MMP-1, and MMP-13 compared to RA-FLS transfected with NC mimics (all *P* < 0.05) (Figure S8A-E). These results aligned with previous findings, confirming that the transfection procedure did not influence the inflammatory response of RA-FLS induced by cBMDM-sEVs.

Furthermore, when cBMDM-sEVs were added to the co-cultures of RA-FLS transfected with miR-100-5p mimics, increased expressions of TNF-α, IL-6, IL-8, MMP-1, and MMP-13 were observed, contrasting with the lower expressions observed in RA-FLS co-cultured with cBMDM-sEVs and transfected with NC mimics. Similar trends were observed with TNF-α stimulation, consistent with the results obtained from IL-1β stimulation (Figure S8F-J). These findings demonstrate that miR-100-5p can effectively reverse the inflammatory-promoting effect of certain cBMDM-sEVs on RA-FLS.

## Discussion

Our study presents significant findings regarding the role of macrophages and EVs in the pathogenesis of RA. RA is a prevalent, chronic, and systemic autoimmune disease characterized by synovitis and autoantibodies. While there has been considerable progress in understanding the interaction between macrophages and RA-FLS, knowledge about the crosstalk between macrophages and RA-FLS in the synovial microenvironment remains limited. Our work demonstrates that BMDM-sEVs exacerbate arthritis under disease conditions. Importantly, the cBMDM-sEVs were found to contain significantly downregulated miR-100-5p, which affects its efficient transport into RA-FLS and consequently inhibits mTOR’s ability to control RA-FLS invasive growth. These findings shed light on biological characteristics of BMDMs under RA conditions that promote disease progression, and provide evidence that macrophages impact the biological function of RA-FLS by secreting abnormal sEVs.

Macrophages are pivotal players in RA pathogenesis, exhibiting a multitude of functions that contribute to inflammation, joint damage, and disease progression [6, 31]. Extensive studies [32–35] have established the role of macrophages in promoting the proliferation and inflammatory expression of RA-FLS, leading to synovial hyperplasia, cartilage erosion, and bone destruction. The interaction between macrophages and RA-FLS forms a feedback loop, with cytokines such as TNF-α and IL-1β derived from macrophages activating FLS, and in turn, FLS promoting macrophage secretion of inflammatory factors. Experimental studies [36–39] also found that depleting macrophages in RA models have shown reduced joint inflammation and destruction, highlighting the detrimental role of macrophages in RA pathogenesis. In this study, our work contributes to the current literature on macrophages in RA by uncovering a novel mechanism involving macrophage-derived sEVs. These sEVs were shown to influence RA-FLS behavior by promoting the release of inflammatory factors and MMPs from RA-FLS and enhancing their proliferation. This evidence provides further support to the notion that macrophages play a crucial role in the communication with RA-FLS, leading to joint inflammation and damage.

Mechanistically, we showed that EVs are responsible for macrophage mediated RA-FLS behaviors. EVs have emerged as key mediators of intercellular communication in various physiological and pathological processes, including autoimmune diseases such as RA. While considerable advancements have been made in understanding the role of EVs in RA, our study adds a unique perspective by investigating the impact of macrophage-derived sEVs on RA-FLS behavior. After in vitro co-culturing of cBMDM-sEVs and nBMDM-sEVs with RA-FLS, BMDM-sEVs from CIA mice were determined to significantly promote both the release of inflammatory factors and MMPs from RA-FLS, and the proliferation of RA-FLS. This result positively correlates with the number of sEVs particles. In vivo cBMDM-sEVs can significantly promote the expression of the inflammatory factors TNF-α, IL-1β, IL-6, and IL-8 in peripheral blood and joint tissues of mice, and the enrichment of MMP-1 and MMP-13 in joint tissues. Consequently, this can promote inflammation and aggravate joint destruction. These results indicate that the precursor cells of macrophages in the disease state are different from those in the normal state and that the sEVs released by them also have different functions. Nevertheless, our study demonstrates that BMDM-sEVs selectively reach inflammatory joints, indicating their potential as targeted therapeutic carriers for RA treatment.

Furthermore, we analyzed miRNA changes in sEVs and identified miR-100-5p as a highly conserved miRNA with immunoregulatory and anti-inflammatory functions. Using miRNA sequencing and qRT-PCR analysis, 11 miRNAs were found to downregulate in cBMDM-sEVs, and miR-100-5p was identified as the most downregulated miRNA in both cBMDM-sEVs and co-cultured RA-FLS. The expression level of miR-146a-5p was lower in the cBMDM-sEVs, as determined by sequencing and qRT-PCR, whereas the expression in the RA-FLS co-cultured with cBMDM-sEVs was inconsistent. We hypothesized that nBMDM-sEVs did not release miR-146a-5p into RA-FLS, or that miR-146a-5p expression may be suppressed in FLS. However, the specific reason remains unclear. miR-100-5p is a highly conserved miRNA with immunoregulatory and anti-inflammatory functions [40]. MSC-enriched miR-100-5p could enhance osteogenesis via modulating mTOR signaling [41]. Another study reported that miR-100-5p overexpression in MSC-exosomes inhibits cell migration and the level of TNF-α, IL-1β, IL-6, and IL-8 in eosinophils [19]. In autoimmune dacryoadenitis, MSC-exosome-enriched miR-100-5p promotes M2 macrophage polarization and Treg generation, thus exerting preventive and therapeutic effects [40]. Luo et al. found that miR-100-5p abundant exosome from MSCs can suppress the expression of IL-6, IL-8, MMP-1, MMP-3, MMP-9, MMP-13, and a disintegrin and metalloproteinase with thrombospondin motifs 5 and protect temporomandibular joint chondrocytes [27]. Similarly, infrapatellar fat pad MSC-derived exosomes enriched with miR-100-5p can partially enhance autophagy in chondrocytes to protect articular cartilage in OA [28]. However, investigations on miR-100-5p in RA are scarce. The present study shows that miR-100-5p overexpression in RA-FLS suppresses proliferation and inflammation, whereas its inhibition promotes inflammation. Moreover, in vivo administration of miR-100-5p agomiR alleviates arthritis in a CIA mouse model, supporting its potential as a therapeutic target.

While our study provides significant insights into the role of macrophages and sEVs in RA, several limitations should be acknowledged. First, we admit the lack of detailed investigation into the specific mechanisms of sEVs assembly and secretion in different RA states. Second, the inconsistent expression of miR-146a-5p in cBMDM-sEVs and RA-FLS co-cultured with cBMDM-sEVs warrants further elucidation. Third, while the study identifies mTOR as an important target of miR-100-5p in RA-FLS, the exact downstream signaling pathways regulated by mTOR in RA-FLS remain to be fully understood. All these limitations highlight the need for additional research to comprehensively address the underlying complexities of macrophage-sEVs interactions and miRNA regulation in RA.

## Conclusion

In conclusion, this study provides valuable insights into the intricate interplay between macrophages and RA-FLS in RA. Our findings reveal that BMDM-derived sEVs play a pivotal role in promoting disease progression by delivering miR-100-5p with immunoregulatory functions that modulate mTOR expression in RA-FLS. These results contribute to the current understanding of the role of macrophages and sEVs in RA pathogenesis, shedding light on potential therapeutic targets.

## Materials and methods

### CIA induction

Herein, 8-week-old DBA/1 mice were obtained from the Beijing Huafukang Biotechnology Company (Beijing, China). Thirty male mice were randomly divided into two groups: CIA group (*n* = 15) and normal group (*n* = 15). The CIA model group underwent the following induction [42]. An intraperitoneal injection of anesthesia and pentobarbital sodium was conducted, after which, chicken-type II collagen (Chondrex, USA) was emulsified to complete Freund’s adjuvant (Chondrex, USA) at a ratio of 1:1, and 100 µL of emulsifier was injected subcutaneously at the root of the tail. After 21 days, an emulsifier of chicken type II collagen and incomplete Freund’s adjuvant were added (Chondrex, USA). Scores were assessed according to the following standards [43]. When the CIA mice developed joint swelling (score ≥ 4), the CIA and normal mice were euthanized to isolate BMDMs. All animal experiments were approved by the Animal Ethics Committee of West China Hospital, Sichuan University (Nos. 2,020,304 A).

### BMDM isolation and culture

BMDMs were isolated from the CIA models and normal mice following previously described methods. The cells were seeded into plates and cultured in a Dulbecco’s modified eagle medium (DMEM) (Gibco, USA), supplemented with 10% heat-inactivated fetal bovine serum (FBS) (Gibco, USA), 1% penicillin-streptomycin solution (Hyclone, USA), and 20 ng/mL recombinant mouse macrophage colony-stimulating factor (M-CSF) (BioLegend, USA) for 3 days.

### sEV isolation and characterization

After the induction of BMDM, the medium was replaced with DMEM containing 10% exosome depleted FBS (SBI, Japan) for 24 h. The medium was centrifuged at 300 g for 10 min, 2 000 g for 20 min, and 10 000 g for 30 min at 4 °C to remove precipitate from the cell supernatant. Subsequently, the supernatant was then ultracentrifuged at 120 000 g for 70 min to collect the pellet. After washing the pellet with PBS, it was resuspended in PBS and again ultracentrifuged at 120 000 g for 70 min, followed by resuspended in 200 µL PBS. The morphology of the sEV was confirmed using TEM (JEM-1400, Japan). The diameter of the sEV was examined using NTA (PMX, Germany). Specific sEV markers were confirmed by WB.

### Cellular uptake assay of BMDM-derived sEVs

cBMDM-sEVs or nBMDM-sEVs were visualized using the PKH67 Green Fluorescent Membrane Linker dye (Sigma, Germany) according to the manufacturer’s instructions. RA-FLS were incubated with PKH67-labed sEVs for 12 h at 37 ℃ with 5% CO_2_. Then cells were washed thrice with PBS and fixed with a paraformaldehyde solution. After blocking for 40 min at room temperature, the α-tubulin antibody (CST 2144, USA) was incubated overnight at 4 ℃. Fluorescent secondary antibodies were incubated with the PBS solution, followed by the addition of 100 µL DAPI. Cells were stained in light for 5 min, the dye solution was discarded, PBS solution was added, and the cells were photographed by fluorescent microscope (Leica, Germany).

### Cell proliferation assays

Approximately 7 × 10^3^ cells were cultured in 96-well plates. After 24, 48, 72 and 96 h, cells were washed twice with PBS and incubated in a 90 µL DMEM with 10 µL of CCK8 reagent (GlpBio, USA). After co-culture for 3 hours, the absorbance at 450 nm were measured using an Enzyme-labelled meter (Bio-Rad, USA).

### qRT-PCR

The total RNA was extracted from the cells using a RNeasy mini kit (Magen, China). RNA from the joint tissue was extracted using TRIZOL (Invitrogen, USA), whereas RNA from the sEVs was extracted using a miRNeasy micro kit (Qiagen, Germany). Reverse transcription of mRNA and miRNA into cDNA was performed using HiScript II RT SuperMix (Vazyme, USA) and the Mir-X miRNA First-Strand Synthesis Kit (Takara, Japan), respectively. ChamQ SYBR qPCR Master Mix and TB Green® Premix Ex Taq ® II (Tli RNase H Plus) were used for qRT-PCR of a CFX96 Real-Time System (Bio-Rad, USA). The mRNA expression was calculated using the 2^−ΔΔCT^ method. GAPDH was used as an internal reference gene. The primers used are listed in Table S1.

### Western blot analysis

Cells and sEVs were lysed using a RIPA lysis buffer (Solarbio, China) containing protease and phosphatase inhibitors (MCE, USA). The protein concentration was determined using a BCA protein assay kit (Beyotime, China). Equal amounts of protein were subjected to SDS-PAGE and transferred to PVDF membranes. The membranes were blocked with a protein free rapid blocking buffer (EpiZyme Biotech, China) and washed thrice with TBST, then incubated with primary antibodies overnight at 4 °C. The primary antibodies used were anti-CD9 (1:1000, Abcam, ab307085), anti-TSG101 (1:1000, Abcam, ab125011), anti-Flotillin (1:4000, Abcam, ab133497), anti-mTOR (1:1000, CST, 2983), p-mTOR (1:1000, CST, 5536), p70s6k (1:1000, Nature bios, A40490), p-p70s6k (1:1000, Nature Bios, A76472), 4E-BP1 (1:1000, CST, 9644), and p-4E-BP1 (1:1000, CST, 2855). The membranes were washed thrice and incubated with an HRP-labeled secondary antibody for 60 min at room temperature. After washing, the membranes were incubated with an enhanced chemiluminescent substrate and visualized using a gel scanner (Bio-Rad, USA).

### Tracking of BMDM-sEVs in vivo

cBMDM-sEVs and nBMDM-sEVs were labeled with 100 μm of DiR dye for 30 min at 37 °C and ultracentrifuged at 120,000 g for 70 min at 4 °C, followed by resuspension in 100 µL of sterile PBS buffer. After measuring the number of sEV particles using NTA, 2 × 10^8^ particles of DiR-labeled cBMDM-sEVs or nBMDM-sEVs were administered to the CIA mice via the tail vein. After 24 h, the mice were euthanized and imaged using an IVIS spectrometer (IVIS, USA). Subsequently, the four limbs were separated and imaged using the same equipment to observe the fluorescence distribution.

### In vivo model of CIA and sEV or miR-100-5p agomiR treatment

After the second immunization, CIA mice were randomly allocated into five groups: PBS, 2 × 10^7^ cBMDM-sEVs group, 2 × 10^8^ cBMDM-sEVs group, 2 × 10^7^ nBMDM-sEVs group, and 2 × 10^8^ nBMDM-sEVs group, with six to seven mice in each group. Tail vein injection began 28 d after the first immunization, with an injection frequency of once per week for three weeks. Different concentrations of BMDM-sEVs were resuspended into 100 µL of sterile PBS solution to be injected into the CIA mice. Similarly, 100 µL of PBS solution was injected into the PBS group. To miR-100-5p experiment, CIA mice were randomly allocated into 2 groups: PBS and agomiR group. Tail vein injection began 28 days after the first immunization, with an injection frequency of twice per week for three weeks. The mice were euthanized 28 days after the first immunization, and the sera and ankles were collected.

### ELISA

The concentrations of TNF-α, IL-1β, IL-6 and IL-8 in the sera were measured using mouse ELISA kits (Elabscience, China) according to the manufacturer’s instructions. The optical density at 450 nm was measured using a microplate reader (Bio-Rad, USA).

### Histological examination

Knee and ankle tissues from the mice were fixed in a 4% paraformaldehyde buffer for 24 h and decalcified for 25 days before being processed for paraffin embedding. Sections were stained with hematoxylin and eosin and subjected to immunohistochemistry (IHC) staining. For the IHC staining, sections were dewaxed, the antigens repaired, blocked with 3% H_2_O_2_ and 5% goat serum, incubated with diluted anti-mTOR overnight at 4 °C, and incubated with horseradish peroxide (HRP)-conjugated secondary antibodies at 37 °C for 1 h. Subsequently, they were developed with DAB solution, stained with citrate, and imaged with a light microscope (Leica, Germany).

### miRNA sequencing

Total RNA was isolated from cBMDM-sEVs and nBMDM-sEVs using the ExoRNeasyE Maxi Kit (Qiagen, Germany) according to the manufacturer’s instructions. Agarose gel electrophoresis (1% gel) was performed to detect RNA degradation and contamination. The RNA purity was determined using a NanoDrop-2000 analyzer. The Qubit ® RNA kit was used to accurately detect the RNA concentration, and the RNA Nano 6000 Assay was used to assess RNA integrity. Library preparation and sequencing were performed by NoveGeneG (Beijing, China) on an Illumina HiSeq XTENXTEN/NovaSeq 6000 sequencer. Data were analyzed using the free online NovoMagic Cloud Platform (www.magic.novogene.com). miRNA-gene interactions were analyzed using TargetScan (http://www.targetscan.org/), miRDB (http://mirdb.org/), and microT (http://diana.imis.athena-innovation.gr/).

### miRNA transfection

When the density of RA-FLS in a 10 cm dish reached 80%, the cells were digested and re-suspended using 400 µL of an electro-transfer buffer (BTX, USA), 20 µL of miR-100-5p mimics or NC-mimics, and 40 µL of miR-100-5p inhibitor or NC inhibitor. The electric rotor cup (BTX, USA) was then placed in the rotate (BTX, USA) under the following conditions: voltage, 200 V; duration, 5 ms; number of pulses, 1; pulse interval, 0.0 s; electrode gap, 2.0 mm. The cells were seeded in 6-well plates. After transfection for 24 h, the cells were harvested for further experiments. miR-100-5p mimics and inhibitors were designed and synthesized by GeneChem (Shanghai, China), and the primer sequences were presented in the Table S2.

### Immunofluorescence

The transfected and non-transfected RA-FLS were seeded in a cell climbing slice in the 12-well plates. After 48 h, the slices were fixed by 4% paraformaldehyde for 10 min, permeabilized using 0.3% Triton X-100 for 10 min, and blocked by 5% goat serum. Cell climbing slices were incubated with rabbit anti-mTOR overnight at 4 °C, washed 3 times by PBS and then incubated with fluorescein secondary antibodies at 37 °C for 1 h. Nuclei were visualized by DAPI (Abcam, USA). Images were captured using an inverted epifluorescence microscope (Leica, Germany).

### Statistical analysis

All data are expressed as mean ± standard deviation and analyzed using GraphPad Prism 8 software (GraphPad Software, CA, USA). Significance was evaluated using an unpaired two-tailed Student’s t-test (two groups) or one-way analysis of variance (ANOVA), followed by Tukey’s multiple comparison tests (multiple groups). Statistical significance was set at *p* < 0.05.

### Electronic supplementary material

Below is the link to the electronic supplementary material.

Supplementary Material 1

## Data Availability

All data are available in the main text or online supplemental materials.

## Abbreviations

RA: Rheumatoid arthritis
FLS: Fibroblast-like synoviocytes
RA-FLS: RA-derived FLS
IL-1β: Interleukin 1β
IL-6: Interleukin 6
IL-8: Interleukin 8
TNF-α: Tumor necrosis factorα
EVs: Extracellular vesicles
sEVs: Small extracellular vesicles
mTOR: Mammalian target of rapamycin
CIA: Collagen-induced arthritis
BMDM: Bone marrow derived macrophage
TEM: Transmission electron microscopy
NTA: Nanoparticle tracking analysis
WB: Western blot
BMDM-sEVs: BMDM-derived sEVs
cBMDM-sEVs: CIA mice derived-BMDM-sEVs
nBMDM-sEVs: normal mice derived BMDM-sEVs
MMP: Matrix metalloproteinases
H&E: Hematoxylin and eosin staining
NC: Negative control
IF: Immunofluorescence
qRT-PCR: Quantitative real-time PCR
p70S6K: p70 ribosomal protein S6 kinase
4E-BP1: 4E-Binding protein 1
KEGG: Kyoto encyclopedia of genes and genomes
GO: Gene Ontology
DMEM: Dulbecco’s modified eagle medium
FBS: Fetal bovine serum
M-CSF: Macrophage colony-stimulating factor
IHC: Immunohistochemistry
