# Supplementary material for: Macrophage-derived mir-100-5p orchestrates synovial proliferation and inflammation in rheumatoid arthritis through mTOR signaling
Source: J Nanobiotechnology. 2024 Apr 22;22:197. doi: 10.1186/s12951-024-02444-1 (PMC11034106; doi:10.1186/s12951-024-02444-1)
Supplement: Supplementary file 1 — Supplementary Material 1 [file 12951_2024_2444_MOESM1_ESM.docx]

**Supplementary Figures and Tables**

**Figure S1.** The mRNA expression of MMP-3 and MMP-9 in the RA-FLS cocultured with PBS, nBMDM-sEVs or cBMDM-sEVs.


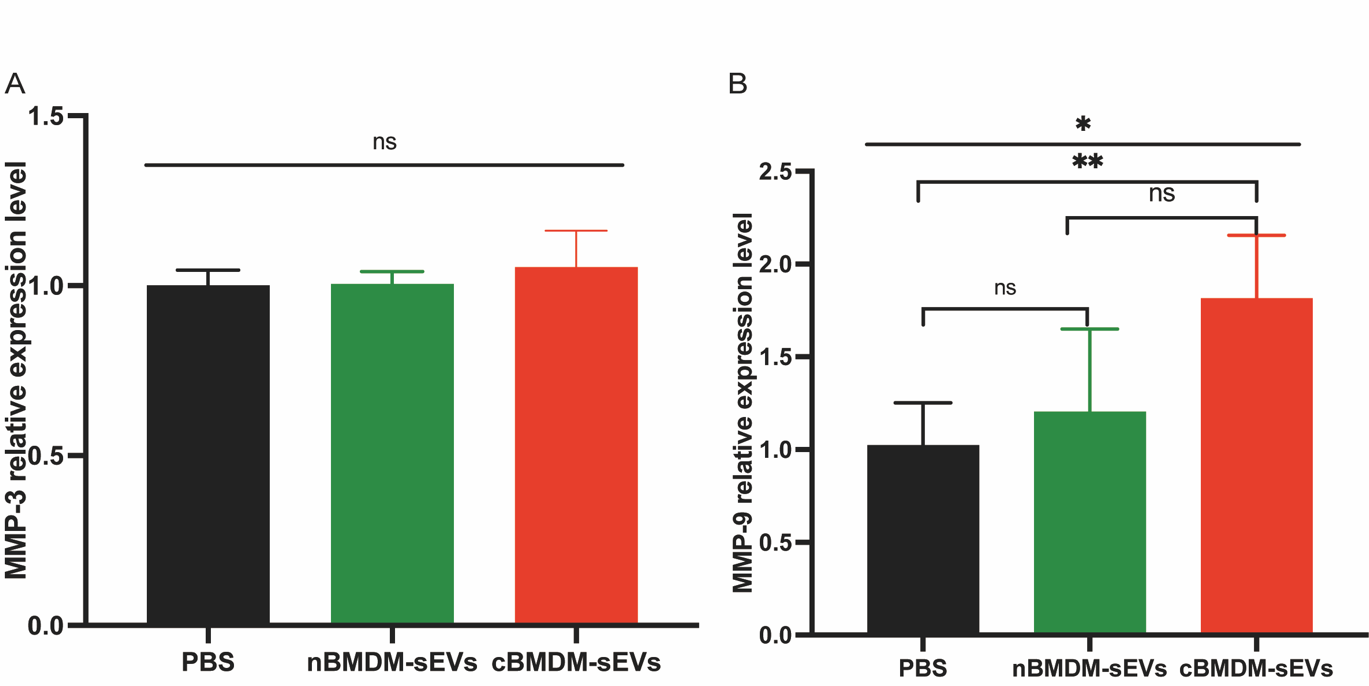


**Figure S2.** The mRNA expression of MMP-3 and MMP-9 in the RA-FLS cocultured with gradient concentration of cBMDM-sEVs.


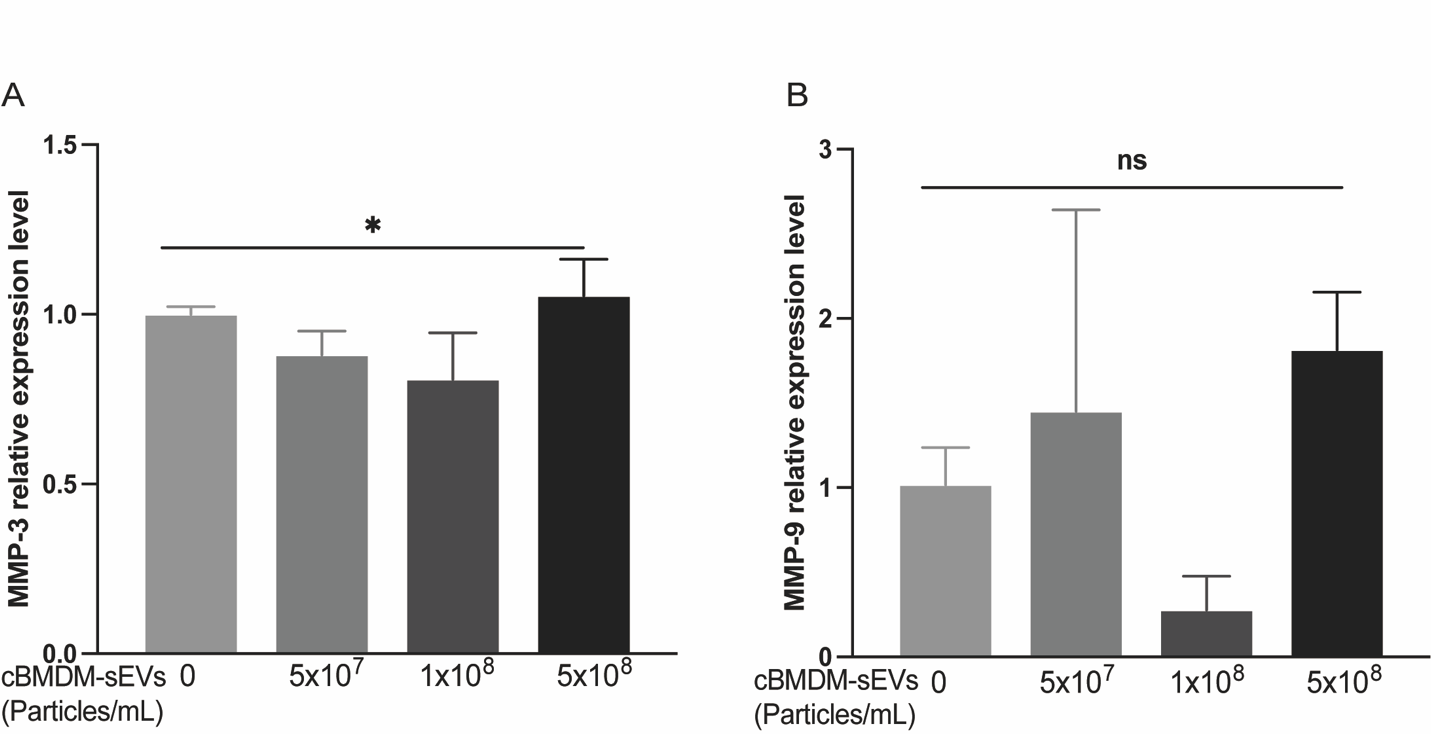


**Figure S3.** Arthritis score in the CIA models, injected with 5×10^7^ particles cBMDM-sEVs, nBMDM-sEVs, respectively.

**Figure S4.** Expression of miR-100-5p in RA-FLS transfected with NC or miR-100-5p inhibitor and stimulated with TNF-α (A) or IL-1β (B).


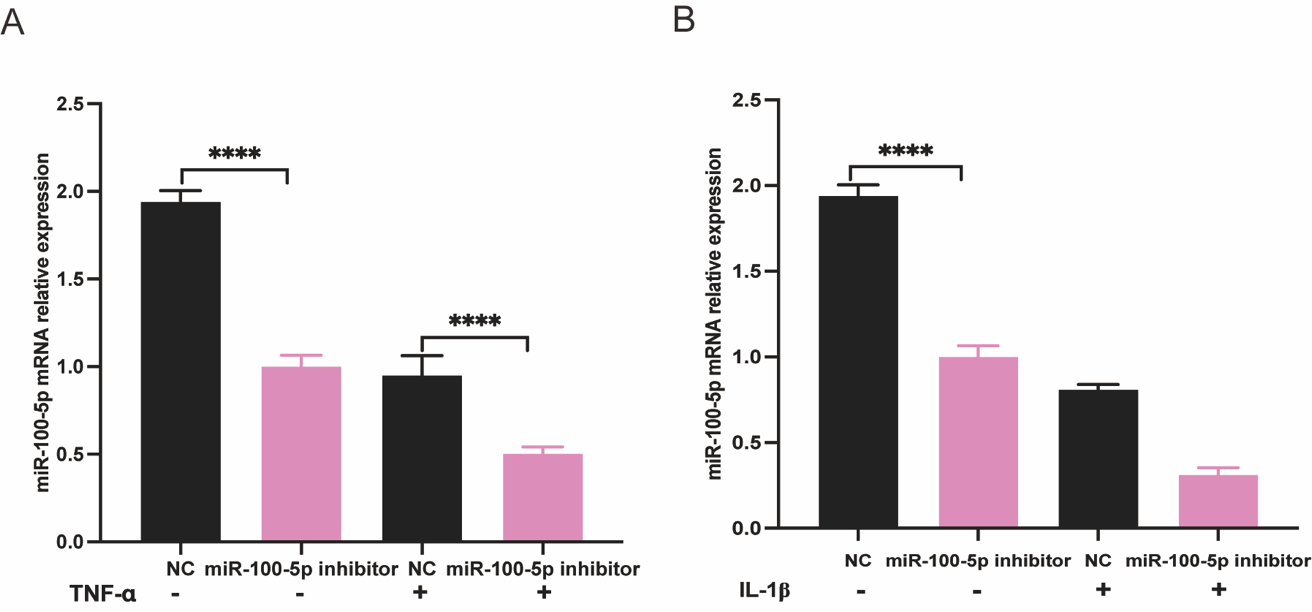


**Figure S5.** Expression of inflammatory cytokines in RA-FLS transfected with NC or miR-100-5p inhibitor and stimulated with TNF-α.

**
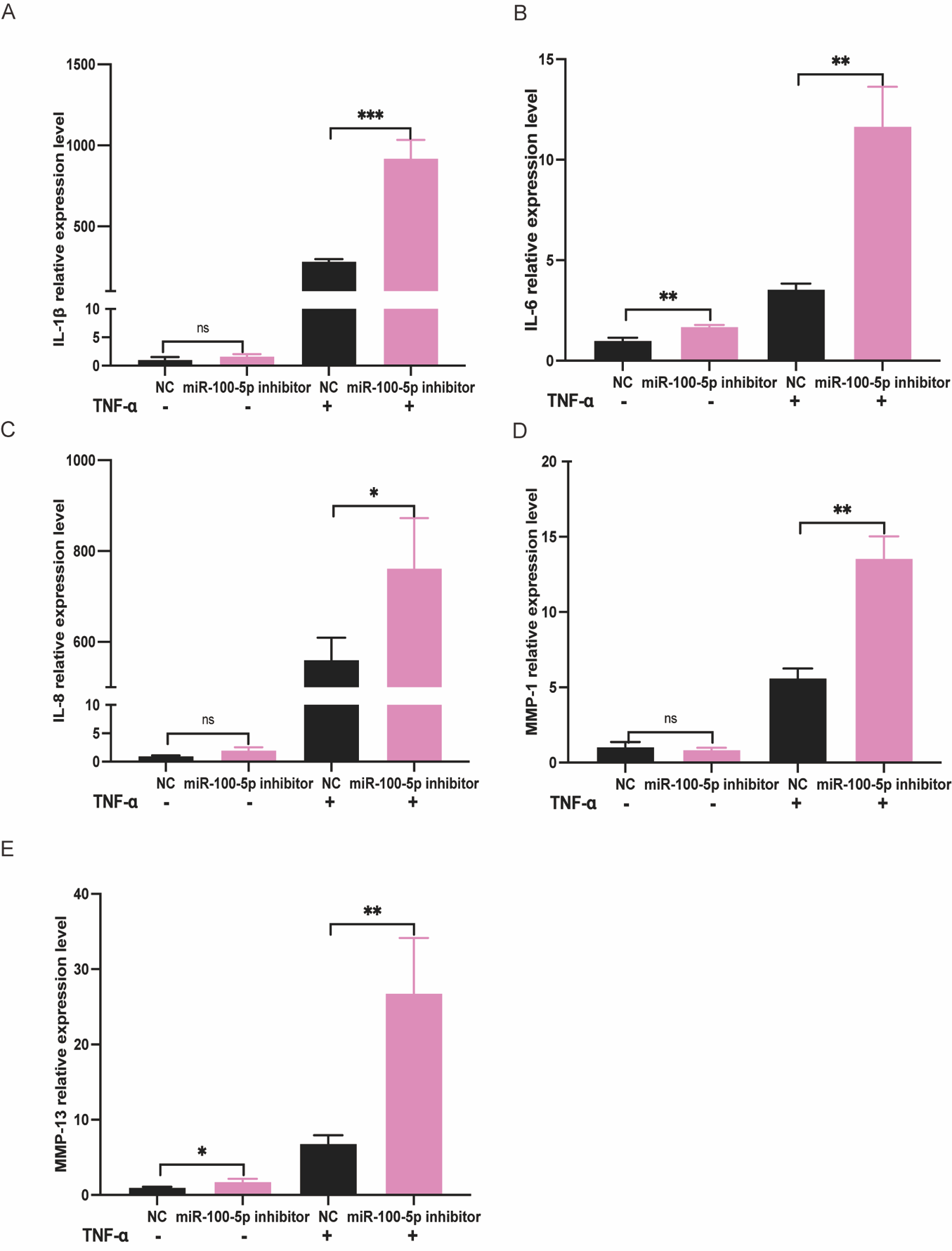
**

**Figure S6.** Expression of inflammatory cytokines in RA-FLS transfected with NC or miR-100-5p inhibitor and stimulated with IL-1β.

**
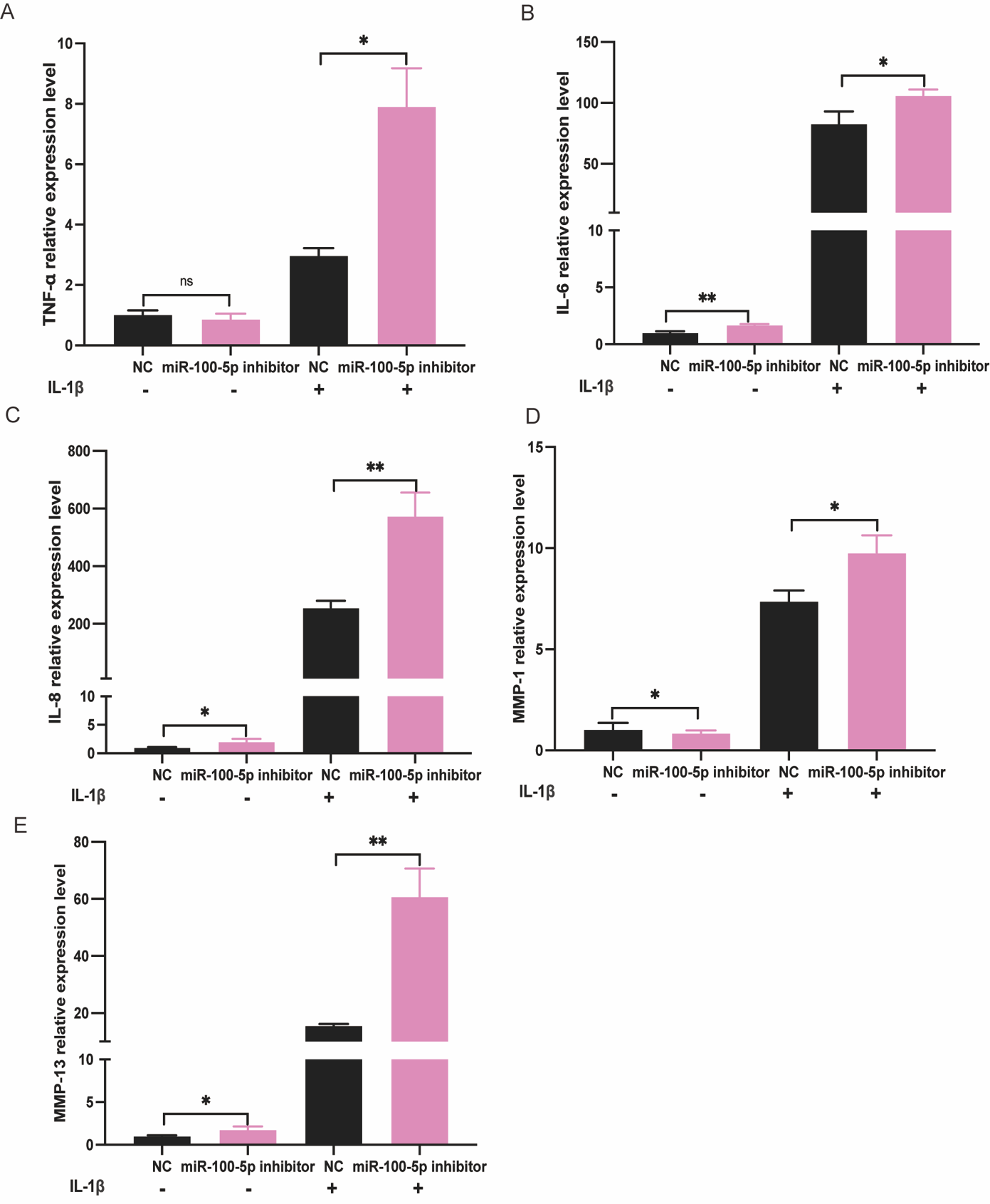
**

**Figure S7.** (A-B) Expression of mTOR in RA-FLS transfected with NC or miR-100-5p inhibitor and stimulated with TNF-α or IL-1β. (C-D) Protein expression of mTORC1 signaling.

**
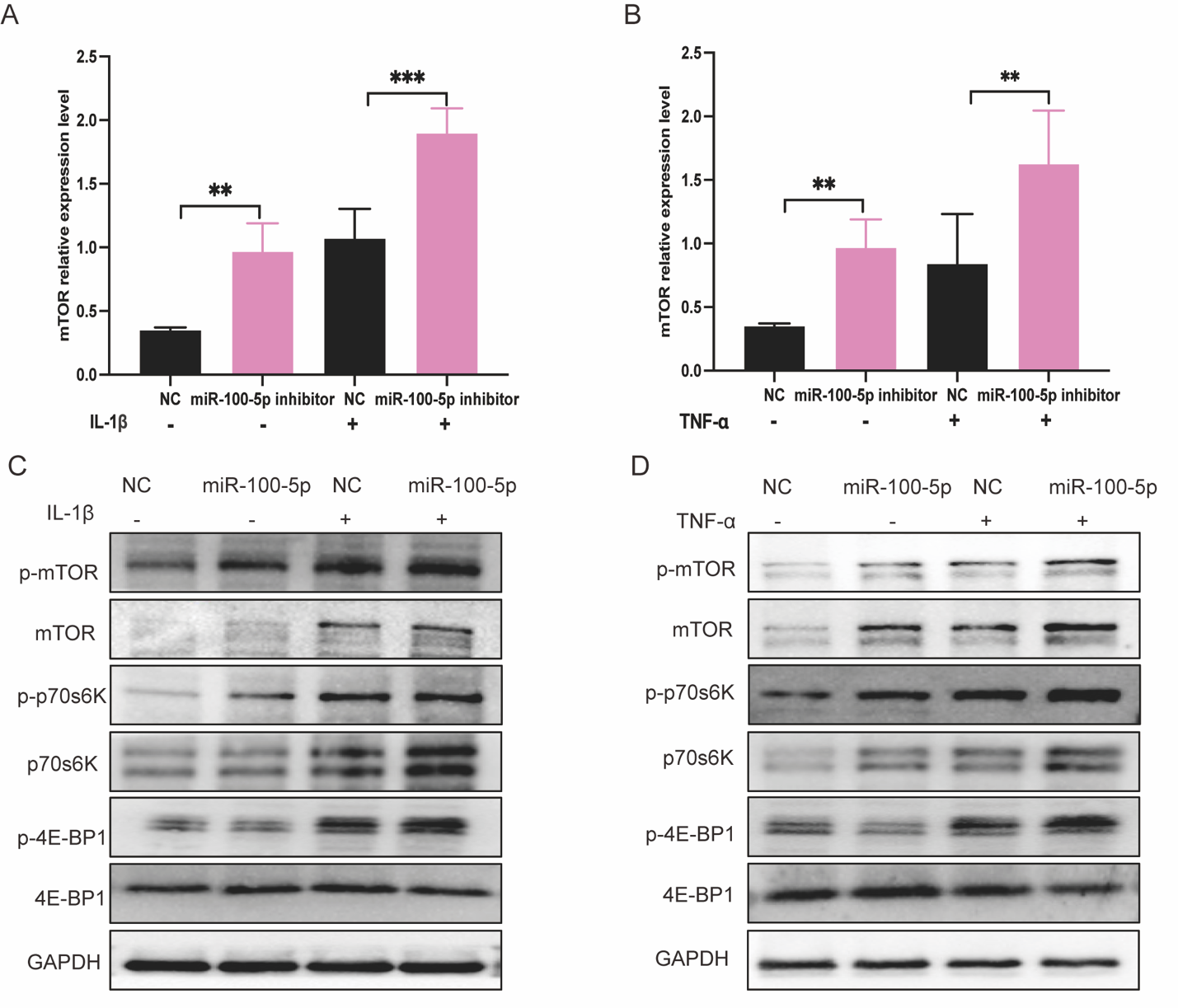
**

**Figure S8.** (A-E) Expression of TNF-α, IL-6, IL-8, MMP-1, and MMP-13 in transfected RA-FLS co-cultured with or without cBMDM-sEVs and stimulated with IL-1β. (F-J) Expression of IL-1β, IL-6, IL-8, MMP-1, and MMP-13 in transfected RA-FLS co-cultured with or without cBMDM-sEVs and stimulated with TNF-α.

**
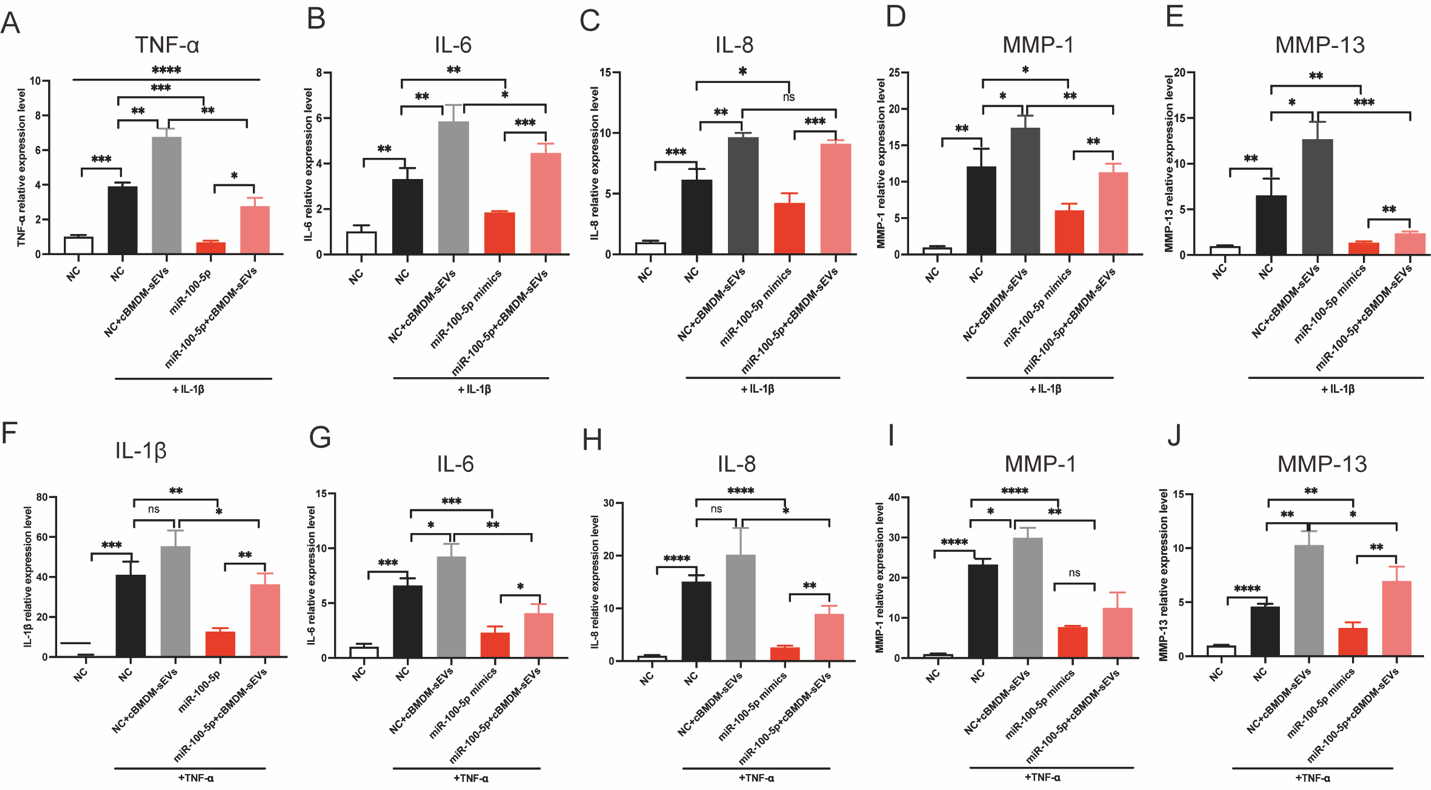
**

**Table S1. qRT-PCR primers in this study.**

|  | **Forward Primers** | **Reverse Primers** |
| --- | --- | --- |
| hIL-1β | TCCAGGAGAATGACCTGAGC | GTGATCGTACAGGTGCATCG |
| hIL-6 | TGAGGAGACTTGCCTGGTGA | TTGGGTCAGGGGTGGTTATT |
| hIL-8 | TGAATGGGTTTGCTAGAATGTG | TGAGGTAAGATGGTGGCTAATAC |
| hTNF-α | CTCTTCTGCCTGCTGCACTTTG | ATGGGCTACAGGCTTGTCACTC |
| hMMP-1 | CACGCCAGATTTGCCAAGAG | GTCCCGATGATCTCCCCTGA |
| hMMP-3 | TGGGCCAGGGATTAATGGAG | GGCCAATTTCATGAGCAGCA |
| hMMP-9 | GGGACGCAGACATCGTCATC | GGGACCACAACTCGTCATCG |
| hMMP-13 | CCTTCCCAGTGGTGGTGATG | CGGAGCCTCTCAGTCATGGA |
| h-mTOR | CACCCTCCATCCACCTCATC | TGTGCTCCAACTCTGTCAAAATC |
| hGAPDH | CACATGGCCTCCAAGGAGTAA | TGAGGGTCTCTCTCTTCCTCTTGT |
| mIL-1β | AATCTCACAGCAGCACATCA | AAG GTGCTCATGTCCTCATC |
| mIL-6 | TTCCATCCAGTTGCCTTCTTG | AGGTCTGTTGGGAGTGGTATC |
| mIL-8 | CCTGCTTGAATGGCTTGAATAC | GGTGTCCTGATTATCGTCCTC |
| mMMP-1 | TTGCCCAGAGAAAAGCTTCAG | TAGCAGCCCAGAGAAGCAACA |
| mMMP-3 | GTTCTGGGCTATACGAGGGC | GGCAGCATCGATCTTCTTCA |
| mMMP-9 | GTTTTTGATGCTATTGCTGAGATCCA | CCCACATTTGACGTCCAGAGAAGAA |
| mMMP-13 | ATGATCTTTAAAGACAGATTCTTCTGG | TGGGATAACCTTCCAGAATGTCATAA |
| m-mTOR | AGAAGGGTCTCCAAGGACGACT | GCAGGACACAAAGGCAGCATTG |
| mGAPDH | AGAACATCATCCCTGCATCC | AGTTGCTGTTGAAGTCGC |

**Table S2. Transfected miRNA mimics or inhibitors in this study.**

|  | **Primers** |
| --- | --- |
| Has-miR-100-5p mimics-cy3 sense | AACCCGUAGAUCCGAACUUGUG |
| Has-miR-100-5p mimics-cy3 antisense | CAAGUUCGGAUCUACGGGUUUU |
| NC mimics-cy3 sense | UUCUCCGAACGUGUCACGUTT |
| NC mimics-cy3 antisense | ACGUGACACGUUCGGAGAATT |
| Has-miR-100-5p inhibitor | CACAAGUUCGGAUCUACGGGUU |
| NC inhibitor | CAGUACUUUUGUGUAGUACAA |
